# Changes in Endogenous Phytohormones of *Gerbera jamesonii* Axillary Shoots Multiplied under Different Light Emitting Diodes Light Quality

**DOI:** 10.3390/molecules27061804

**Published:** 2022-03-10

**Authors:** Monika Cioć, Michał Dziurka, Bożena Pawłowska

**Affiliations:** 1 Department of Ornamental Plants and Garden Art, Faculty of Biotechnology and Horticulture, University of Agriculture in Kraków, 29 Listopada 54, 31-425 Kraków, Poland; bozena.pawlowska@urk.edu.pl; 2 Department of Developmental Biology, The Franciszek Górski Institute of Plant Physiology, Polish Academy of Sciences, Niezapominajek 21, 30-239 Kraków, Poland; m.dziurka@ifr-pan.edu.pl

**Keywords:** stress-related hormones, UHPLC, light emitting diodes, in vitro, auxins, gibberellins, cytokinins, phytohormonal balance, metabolic pathways, growth regulators

## Abstract

Light quality is essential in in vitro cultures for morphogenesis process. Light emitting diodes system (LED) allows adjustment as desired and the most appropriate light spectrum. The study analyzed the influence of different LED light quality on the balance of endogenous phytohormones and related compounds (PhRC) in in vitro multiplied axillary shoots of *Gerbera jamesonii*. Over a duration of 40 days, the shoots were exposed to 100% red light, 100% blue light, red and blue light at a 7:3 ratio with control fluorescent lamps. Every 10 days plant tissues were tested for their PhRC content with the use of an ultra-high performance liquid chromatography (UHPLC). Shoots’ morphometric features were analyzed after a multiplication cycle. We identified 35 PhRC including twelve cytokinins, seven auxins, nine gibberellins, and seven stress-related phytohormones. Compounds content varied from 0.00052 nmol/g to 168.15 nmol/g of dry weight (DW). The most abundant group were stress-related phytohormones (particularly benzoic and salicylic acids), and the least abundant were cytokinins (about 370 times smaller content). LED light did not disturb the endogenous phytohormone balance, and more effectively mitigated the stress experienced by in vitro grown plants than the fluorescent lamps. The stress was most effectively reduced under the red LED. Red and red:blue light lowered tissue auxin levels. Blue LED light lowered the shoot multiplication rate and their height, and induced the highest content of gibberellins at the last stage of the culture.

## 1. Introduction

*Gerbera jamesonii* is an ornamental species from the Asteraceae family, belonging to the group of the most economically important plants. It is one of the most popular and valued commercial cut flowers, ranked fifth in the world (after roses, carnations, chrysanthemums, and tulips) [1]. In the years 2010–2019, Canada, Mexico, Indonesia and Japan jointly produced 76 million cut gerbera stems and four million indoor potted gerberas [2]. The Netherlands produces 420 million gerbera cut flowers annually, the value of which is estimated to be around 67 million EUR [3]. The gerbera is also one of the most popular cut flowers in France, Spain and Italy [4].

The starting material for gerbera production is sourced exclusively from in vitro cultures, where microplants of high quality are produced with great efficiency [5,6]. The key goal is to obtain the best quality microplants that would successfully develop into marketable final products. An important challenge faced by the breeders is to lower the production costs without compromising quality. The use of light emitting diodes (LEDs) is an environmentally-friendly solution that uses less electricity and does not yield toxic waste, as was the case with fluorescent lamps (Fl). LED lighting is up to 95% more efficient than traditional lighting used in phytotrons [7,8,9]. The external conditions of an in vitro culture, including the quality of light, affect plant growth and development. The spectral composition of light controls the morphogenesis, synthesis of organic compounds, and changes at the biochemical and physiological levels [10]. The environmental stimuli target the biosynthesis or perception of phytohormones, thus coordinating internal developmental programs [11]. Endogenous phytohormones are synthesized in plant tissues. They are organic substances that trigger physiological processes such as growth, differentiation and development, or, for example, opening of the stomata (at low concentrations) [12,13]. They are chemical messengers, which do not play a single biological role in plants, but play various roles at different stages, in different tissues or under different environmental conditions [14]. There are many important and well-known plant hormones such as auxins, abscisic acid, cytokinins, gibberellins (GAs) and also ethylene, brassinosteroids, salicylic acid, jasmonates, and strigolactones [15,16,17]. The term plant growth regulators (PGR) is an umbrella term for exogenous natural compounds or their synthetic analogs that act as natural plant hormones [18]. They directly affect the plant phytohormonal balance [19,20,21]. The research literature contains few papers on endogenous regulating substances present in plant tissues during various processes and stages of in vitro development together with the impact of LED light quality, and those interactions at the hormonal pathways level (e.g., [22,23]). Moreover, many publications still investigate only the role and effect of externally supplied regulating substances (in the media) on plant growth and development.

Light, as environmental factor, is a key player in plant morphogenesis. It regulates plant growth and development, and also influences the hormone levels [24,25]. The effects of light quality on these specific physiological and biochemical responses are much more complex than those caused by its intensity or photoperiod [26]. Light of a different quality, applied during shoot multiplication, affects morphogenetic processes, albeit indirectly [27,28,29]. For example, red and blue light combination promotes a higher number of adventitious shoots of *Anthurium andraeanum*, and the red light alone makes their shoots longer [30]. Red light also promotes the proliferation rate of *Stevia rebaudiana* [31]. Blue light can promote the number of leaves produced by a rose [32] and the greatest callus differentiation with the highest total multiple shoots of *Vanilla pompano* [33]. Light signals are transmitted from photoreceptors and induce gene expression, ultimately resulting in those physiological responses and developmental changes. Many hormonal pathways are often modulated by light. Moreover, phytohormones are involved in plant responses to light [34,35]. The light and hormonal signals can interact, for example, to control the growth of the hypocotyl in *Arabidopsis thaliana* seedlings [34]. Integrators connecting the light and hormone signaling pathways have been identified; such as PHYTOCHROME-INTERACTING FACTOR 3 (PIF3), PIF4, PIF3-LIKE 5 (PIL5)/PIF1 and LONG HYPOCOTYL 5 (HY5) [35]. The balance of endogenous phytohormones involved in the regulation of photomorphogenesis probably depends on the spectral composition of light [34].

The research described here focused on the multiplication of the axillary shoots of a *Gerbera jamesonii* ‘Big Apple’ under LED light of different quality. Our studies analyzed the changes in the content of endogenous phytohormones and other phytohormone related compounds (PhRCs) acting as signal substances that affect the course of changes and plant development during this process. Despite existing similar research on the topic (e.g., [36]), our research concerns pay attention to the participation of a different quality of LED light in this aspect. Different responses of the gerbera plants are referred to the PhRC potential metabolic pathways and different stages of in vitro multiplication. The study aims to determine the influence of different light quality on the balance of endogenous phytohormones and related compounds (PhRC) in the tissues of multiplied gerbera shoots. The analyses were carried out from the moment the explants were placed into the medium (starting the culture) until the culture termination (40 days), and were compared with multiplication efficiency and plant quality, as determined by biometric parameters.

In this study we analyzed the plant tissue response to a different quality of light at different stages of axillary shoot culture. The obtained results broaden our knowledge of how light quality influences the changes in PhRC content during in vitro plant cultivation, with regard to their metabolic pathways and plant morphometric response. In the experiment an economically justified LED lighting system was used [37,38,39,40]. Red and blue light spectrum are the most important for plants, due to the regulatory effect on their development, which is connected to the chlorophyll absorption peaks [28,41,42]. In the experiment red and blue light impact was tested separately (basic research) and in combination, as recommended for many species and varieties of plants in in vitro cultures [30,39,43,44]. The obtained results will complement the current knowledge on the effects of these wavebands.

## 2. Results

**Figure 1:**
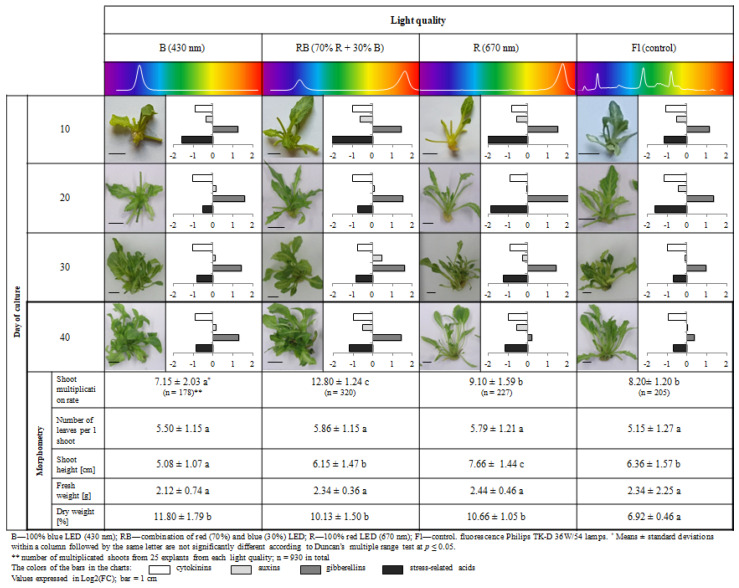
Total content of cytokinins, auxins, gibberellins and stress related hormones at different stages of the culture (after 10, 20, 30 and 40 days) versus their content at the beginning of the culture (day zero), and morphological parameters of the plantlets and their weight after 40 days of multiplication. B—100% blue light-emitting diode (LED); RB—mixed red and blue LED (7:3); R—100% red LED; Fl—control, white fluorescent light; scale bar = 1 cm, fold change of phytohormones in comparison with the starting material values are log2 transformed.

**Figure 2:**
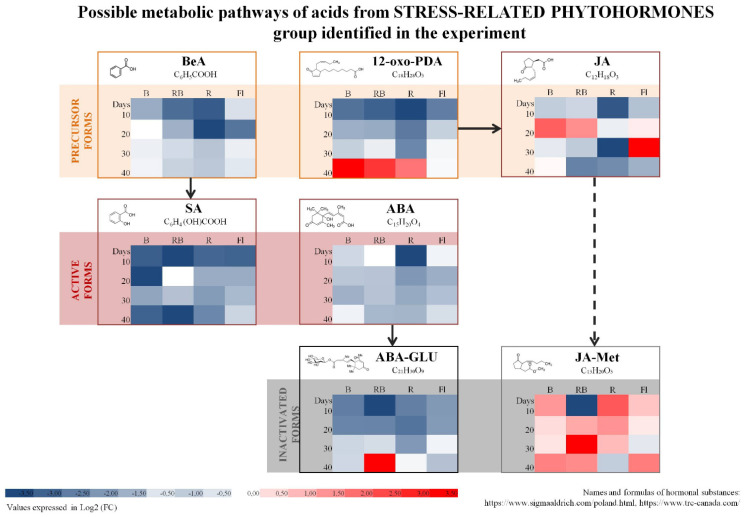
Schematic diagram of stress-related hormones and related compounds with heat maps representing changes in the accumulation of particular compounds during the experiment. Values of fold change of phytohormones in comparison with the starting material are log2 transformed. Coloring intensity represents the relative value, red shades represent accumulation, whereas blue shades represent relative content decrease. Benzoic—benzoic acid, 12-oxo-PDA—12-oxo-phytodienoic acid, SA—salicylic acid, JA—jasmonic acid, ABA—abscisic acid, JA-Met—jasmonic acid methyl ester, ABA-GLU—abscisic acid glucosyl ester. Metabolic pathway scheme according to: [46,47,48].

**Figure 3:**
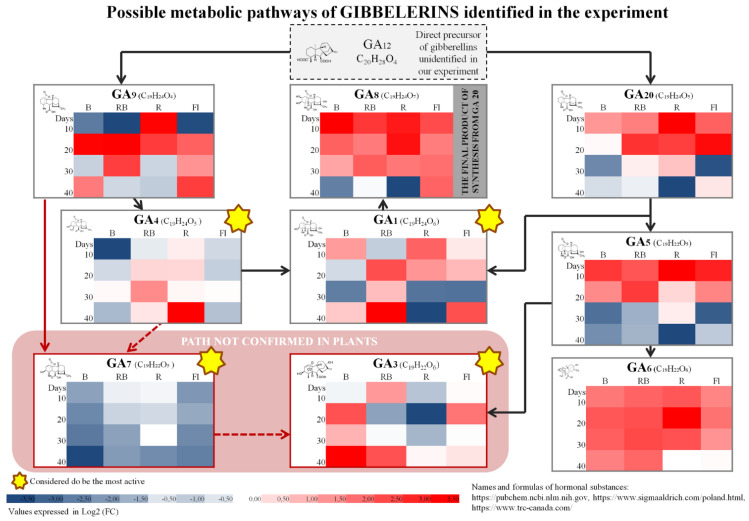
Schematic diagram of gibberellin metabolic pathways with heat maps representing changes in the accumulation of particular compounds during the experiment. Values of fold change of phytohormones in comparison with the starting material are log2 transformed. Coloring intensity represents the relative value, red shades represent accumulation, whereas blue shades represent relative content decrease. Metabolic pathway scheme according to: [45,49,50].

**Figure 4:**
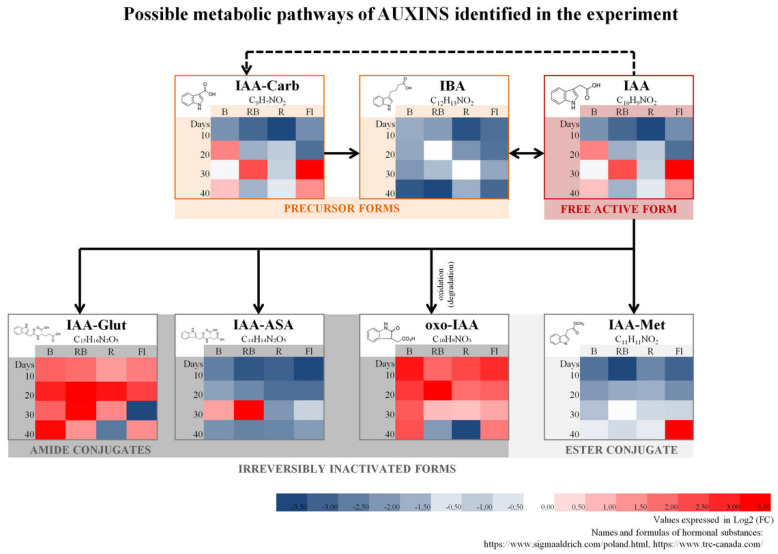
Schematic diagram of auxin metabolic pathways with heat maps representing changes of particular compound accumulation during the experiment. Values of fold change of phytohormones in comparison with the starting material are log2 transformed. Coloring intensity represents the relative value, red shades represent accumulation, whereas blue shades represent relative content decrease. IAA-Carb—indole-3-carboxylic acid, IBA—indole-3-butyric acid, IAA—indole-3-acetic acid, IAA-Glut—indole-3-acetyl-l-glutamic acid, IAA-ASA—indole-3-acetyl-l-aspartic acid, oxo-IAA—oxindole-3-acetic acid, IAA-Met—indole-3-acetic acid methyl ester. Metabolic pathway scheme according to: [51,52,53,54].

**Figure 5:**
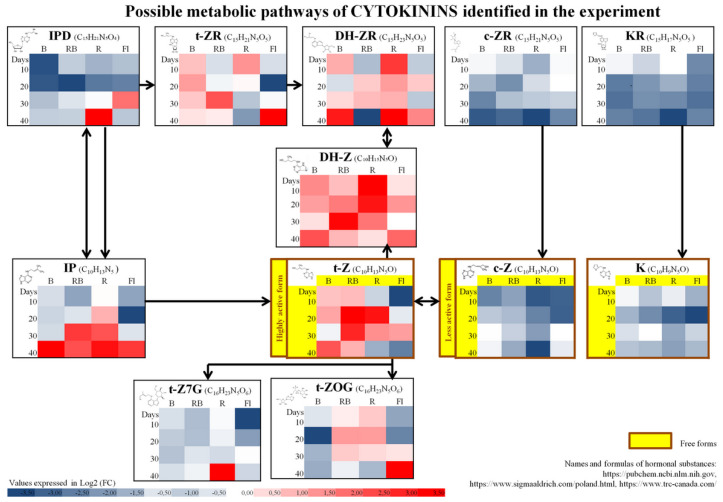
Schematic diagram of cytokinin metabolic pathways with heat maps representing changes of particular compound accumulation during the experiment. Values of fold change of phytohormones in comparison with the starting material are log2 transformed. Coloring intensity represents the relative value, red shades represent accumulation, whereas blue shades represent relative content decrease. IPD—N6-isopentenyladenosine, t-ZR—trans-zeatin riboside, DH-ZR—dihydrozeatin riboside, c-ZR—cis-zeatin riboside, KR—kinetin riboside, DH-Z—dihydrozeatin, IP—N6-isopentenyladenine, t-Z—trans zeatin, c-Z—cis zeatin, K—kinetin, t-Z7G—trans-zeatin-7-glucoside, t-ZOG—trans-zeatin-o-glucoside. Metabolic pathway scheme according to: [55,56,57,58,59,60,61].

The study involved multiplication of axillary shoots of gerbera ‘Big Apple’ under different lighting conditions. We used four sources of light: B—100% blue LED (430 nm), RB—combination of red (70%) and blue (30%) LED, R—100% red LED (670 nm), Fl—control, fluorescent lamps. Samples collected during multiplication every 10 days confirmed the presence of 35 endogenous phytohormones and related compounds (PhRC) belonging to cytokinins (12), auxins (7), gibberellins (9), and stress-related phytohormones (7) (Figure 1, Figure 2, Figure 3, Figure 4 and Figure 5). For the identified phytohormones, the potential metabolic pathways of the above-mentioned PhRC groups were plotted to show the formation of the phytohormonal and metabolic balance (Figure 2, Figure 3, Figure 4 and Figure 5). For each PhRC, we also present heat maps visualizing changes in PhRC content in relation to the starting material for the consecutive stages of the culture. Red color on the heat map indicates increased and blue decreased PhRC content, while the color intensity marked by the gradient created by varying the lightness of a single hue indicates the size of the differences versus the original values.

The level of PhRCs varied widely from 0.52 pmol/g (*cis*-zeatin, c-Z) to 168.15 nmol/g of dry weight (DW) (benzoic acid, BeA), so we decided to present the PhRC results on a logarithmic scale as a fold change versus accumulation in the starting material (control). This is visible in Figure 1, which also shows the stages of morphological development of the axillary shoots after 10, 20, 30 and 40 days of the culture, and the morphometric parameters determined at the end of the culture.

In the starting material, the stress-related phytohormones accounted for over 95% of all phytohormones (Table 1). The hormones least abundant during growth were cytokinins. Their mean content ranged from 0.38 to 0.49 nmol/g, irrespective of the light quality and stage of the culture (Table S1). Auxins and gibberellins were on average twenty times more abundant (5.34 to 11.15 nmol/g and 3.91 to 15.83 nmol/g, respectively). Most of the identified PhRCs were stress-related phytohormones, which accounted for almost 90% of all phytohormones identified during multiplication. Their content ranged on average from 77.43 to 247.63 nmol/g. This tendency was maintained at all stages of the culture and all tissues derived from in vitro plants, regardless of the light quality. The only exception to this rule were the plants growing under red light between the 10th and 20th day of the culture, where the content of stress-related phytohormones was lower (over 80%). However, this variant exhibited an increased content of gibberellins (Table S1, Figure 1).

Seven stress-related phytohormones were identified: salicylic acid (SA) and its precursor—benzoic acid (BeA); jasmonic acid (JA), its precursor 12-oxo-phytodienoic acid (12-oxo-PDA) and its ester form jasmonic acid methyl ester (JA-Met); abscisic acid (ABA) and its inactivated form—abscisic acid glucosyl ester (ABA-GLU). Figure 2 presents metabolic dependencies and a heat map representing relative changes in their accumulation during culture. Red LED light (R and RB) most effectively reduced the content of stress-related phytohormones, and after 10 days the fold change decrease was over four (Figure 1). Throughout the entire period of shoot multiplication, the content of majority of the stress-related phytohormones was lower than in the starting material. Among the identified PGRs belonging to stress-related phytohormones, the most abundant were salicylic acid (average content 45.10 nmol/g) and its precursor benzoic acid, the average content of which reached 104.88 nmol/g. These substances constituted the major part of total stress-related phytohormones (Table S1).

With time and consecutive stages of the shoot multiplication the content of benzoic acid gradually rose. Under B and Fl light it reached the level similar to that in the starting material. A contrary trend was observed for its active form of salicylic acid, whose content dropped under LED (B, R and RB) light. Another compound of considerable levels in this group was jasmonic acid (on average 5.70 nmol/g), while abscisic acid was about 500 times less abundant than benzoic acid. Stress-related phytohormone with the lowest content was jasmonic acid methyl ester (JA-Met) (on average 0.04 nmol/g), however, at nearly all stages of the culture its level was higher than in the starting material. At the early stages, the content of 12-oxo-phytodienoic acid was lower than in the starting material, although at the end of the culture it grew in the plants exposed to all combinations of LED light. Its average content in the starting material was 11.43 nmol/g, and after 40 days under LED light, it reached on average 15.02 nmol/g.

The detected gibberellins (GAs) included: GA_5_, GA_6_, GA_8_, GA_9_, GA_20_. Those considered the most active were: GA_1_, GA_3_, GA_4_ and GA_7_ [45]. The total content of all gibberellins during the culture was always higher than in the starting material (Figure 1), irrespective of the light quality. Figure 3 shows specific changes in gibberellin content, and the trend is visible for most of the substances. The only exception was GA_7_, whose content at all stages of shoot multiplication did not exceed that in the starting material. We also identified a general trend of dropping gibberellin levels toward the end of the culture (after 40 days), particularly in plants grown under red LED (R) and fluorescent (Fl) light (Figure 1). For GA_7_ and GA_5_ it was even lower than in the starting material (Figure 3). The most abundant among the identified gibberellins was GA_6_ (on average 8.00 nmol/g), and it was the decisive factor affecting the total changes in the levels of this PhRC group.

We detected seven substances belonging to auxins (Figure 4), including: a precursor form—indole-3-butyric acid (IBA), indole-3-carboxylic acid (IAA-Carb) and free active form—indole-3-acetic acid (IAA), as well as its various inactivated forms: oxindole-3-acetic acid (oxo-IAA), indole-3-acetyl-l-aspartic acid (IAA-ASA), indole-3-acetyl-l-glutamic acid (IAA-Glut), and indole-3-acetic acid methyl ester (IAA-Met). After 10 days of the culture, the total level of auxins dropped in relation to the starting material by about 1.5 times (Table S1, Figure 1). However, in the next phase of the culture, after 20 days of shoot multiplication, their content increased, and under LED light containing the blue wavelength (B and RB) it was higher than in the starting material (Figure 1). After 30 days, the plants grown under red LED light (R) responded with a decrease in total auxin content. Finally, after 40 days, the drop also occurred under RB LED mixed light. These results suggest that red light spectrum (R and RB LED), along with the duration of the culture and multiplication of axillary shoots, reduce the total level of auxins in the tissues, and the effect was perceived earlier for R LED light than for the mixed RB LED spectrum. This drop in auxin levels was not observed for B LED or control Fl. Detailed analyses of the level of individual auxins over the culture duration demonstrated specific changes depending on the light quality.

Under control fluorescent light, we witnessed an increase in the content of all auxins except for oxindole-3-acetic acid, whose levels dropped, although they were still higher than in the starting material. 

Under red LED light (R) the content of endogenous auxins was always lower than in the starting material, except for inactivated forms of indole-3-acetyl-l-glutamic (IAA-Glut) and oxindole-3-acetic acids (oxo-IAA). 

The content of the determined auxins ranged from 0.31 to 2.50 nmol/g, unlike in the other PhRC groups. The least abundant auxins were the inactivated indole-3-acetic acid methyl ester (IAA-Met) and the precursor—indole-3-butyric acid (IBA) (on average 0.33 nmol/g). Contrary to that, we measured considerable levels of the active auxin IAA (1.33 nmol/g) and its inactivated forms IAA-Glut and IAA-ASA (respectively, 1.23 and 1.36 nmol/g). The most abundant auxin was the precursor indole-3-carboxylic acid (IAA-Carb).

In the group of the least abundant endogenous phytohormones—cytokinins, we identified 12 different compounds (Figure 5), classified based on the configuration of their side chain as isoprenoids: N_6_-isopentenyladenosine (IPD), N_6_-isopentenyladenine (IP); isomeric forms *cis* and *trans* zeatin (t-Z, c-Z) and their ryboside forms: trans-zeatin riboside (t-ZR), cis-zeatin riboside (c-ZR), and glucoside forms: trans-zeatin-7-glucoside (t-Z7G), trans-zeatin-o-glucoside (t-ZOG); dihydrozeatin forms: dihydrozeatin riboside (DH-ZR), dihydrozeatin (DH-Z); aromatic forms of kinetin: kinetin riboside (KR), kinetin (K). Of those, the lowest amounts were detected for an active form of zeatin—cis zeatin (on average 0.00089 nmol/g). The content of other active forms reached average levels (from 0.024 for trans zeatin to 0.030 nmol/g for kinetin).

The mean total content of all identified forms of cytokinins at all stages of the culture and for all light qualities was lower than in the starting material (Figure 1). This trend was followed by active forms of c-Z and K, while t-Z levels under B and RB LED were higher than in the starting material over the entire culture, and under R and Fl they dropped below the initial value on the 40th day of the culture. The contents of the remaining identified cytokinins varied depending on the stage of the culture and the light quality, and they were not always below the content in the starting material, as shown by the general summary trend (Figure 5). For example, dihydrozeatin (DH-Z) was more abundant at all stages of the culture and for all light quality treatments, and N_6_-isopentenyladenine (IP) also accumulated in greater amount at the end of the culture. The highest average content was determined for dihydrozeatin riboside (DH-ZR) (0.24 nmol/g), which was also more abundant in plant tissues at the end of the culture than in the starting material, except for mixed LED (RB) variant.

Figure 1 presents morphogenetic response of the plants at the end of the shoot multiplication culture. The shoots grown and multiplied under LEDs developed properly. Mixed RB LED stimulated their multiplication rate, as it was nearly two times higher than under blue LED and reached 12.8. The value was similar under red LED (R) and control fluorescent lamp (Fl) (ca. 8–9 plants), and the plants growing under R LED light produced the highest shoots. Light quality did not affect the number of leaves and new shoots or fresh weight of the multiplied shoots. The shoots growing under control fluorescent lamp (Fl) reached the lowest dry weight, as it was 32–41% lower than under the LEDs. Our study confirmed the effectiveness of LEDs in axillary shoot multiplication of ‘Big Apple’ gerbera.

## 3. Discussion

### 3.1. LED Light Impact on Gerbera Multiplication

The multiplication of gerbera shoots under the LEDs ensures their proper growth and development. The plants also maintain an acceptable morphometric and physiological status. LEDs are highly efficient in the multiplication of ‘Big Apple’ gerbera shoots. Light stress is the less studied among the different types of abiotic stresses experienced by plants [62]. We found no visible stress symptoms, like growth inhibition or abnormal development, concerning light quantity and quality, even on the phytohormonal level, and LED light even improved some parameters when compared with fluorescent lamps. Positive effects of LED light on gerbera cultures were reported in our earlier studies [28,29,40,63] and in a paper by Meng et al. [64], where mixed RB LED light (7:3) stimulated gerbera growth in vitro and improved the physiological condition of the plants. As demonstrated in these studies: the multiplication rate, as well as the content of photosynthetic pigments, was increased [28] and leaf quality was better [44], as shown in our earlier studies. Furthermore, the mixture of red and blue LED light in a 7:3 proportion positively influenced the functioning of the photosynthetic apparatus and did not reduce the secondary metabolism, on the mono- and oligosaccharide level, content of gerbera plants [44]. The use of LED light also caused an increased accumulation of many flavonoid compounds in *Cyathea delgadii* in vitro culture, compared to Fl light [65]. Gök et al. [66] concluded that treatment with blue (100%), white (100%) and mixed RB (7:3) LED light could provide a viable alternative to white fluorescent lamps in micropropagation of gerbera ‘Rosalin’. Positive effect of the use of LED lamps is visible even at the dry weight level. In our studies it was greater in plants multiplied under LEDs compared to those multiplied under florescent lamp light. For plant growth promotion tests, dry weight determination is recommended rather than fresh weight determination [67]. The plantlets obtained by us do not differ statistically when it comes to fresh weight, which may indicate a greater hydration of their tissues (plants from Fl light). 

The outcomes of studies on additional illumination of gerbera produced for cut flowers in greenhouses also look promising. Zheng et al. [68] obtained cut gerbera flowers of better quality under LEDs than HPS lamps. Microcuttings for such a production can be multiplied in vitro under LEDs, as their hormonal status does not indicate any greater stress than under fluorescent lamps, as shown in our study, than what we observe in stress-related phytohormones accumulation. Moreover, such conditions provided quicker abatement of the stress symptoms typical of in vitro culture at the beginning of the multiplication cycle. This stress is caused, among others things, by mechanical injuries, wounding, osmotic shock (high sucrose content in the medium), hormonal imbalances and so on. The plantlet is transferred to new growth conditions, provided by an in vitro culture. A growing and developing plant organism is forced to adapt to these new conditions, and alter the physiology and morphology of plantlets [69].

### 3.2. The Role of Plant Growth Regulators

The analyses carried out in this work are the first to describe the effects of LED light quality on changes in endogenous hormones and related compounds (PhRC), and comprehensively report the course of these changes in gerbera tissues at the subsequent stages of in vitro multiplication of axillary shoots. The substances identified in the study were classified into four groups: cytokinins; auxins; gibberellins; and stress-related phytohormones. Papers available in the research literature mainly focus on the effects of PGR added to a medium, sometimes in combination with light, on in vitro morphogenesis. In the presence of exogenous growth stimulators, the indirect role of light may be obscured [70]. However, exogenous PGRs are necessary to stimulate various developmental and physiological processes. Some species require an exogenous cytokinin to grow properly [71], as also confirmed in our earlier studies [40]. Contrary to that, other papers suggest complementarity between red light and exogenous cytokinin. In some cases (*Spiraea*—supplementation with BA, *Lemna gibba*—supplementation with kinetin), red light can replace exogenous cytokinin or enhance its effects on plants by specifically affecting growth or morphogenesis in vitro and promoting axillary shoot growth [70,72,73]. However, different species may respond differently and there are also reports on similar effects on blue light, e.g., in *Vitis* (supplementation with BA) [74]. Growth and development in in vitro cultures are controlled by interaction and balance between exogenously supplied and endogenously produced plant growth regulators [71]. The externally supplied PGRs can modify the synthesis, destruction, activation, sequestration, transport or sensitivity to endogenous growth substances of the same or a different type [18]. In our study, we confirmed this correlation for cytokinins.

### 3.3. Stress-Related Phytohormones 

In vitro organogenesis can be divided into consecutive growth stages of a plantlet, according to the stages of cultured cells in an incubated environment. These are; lag phase, period of exponential, linear and logarithmic growth, then the phase of decreasing growth, stationary phase, culture unviability, and finally death phase [70,75]. Gerbera shoot multiplication culture was terminated after 40 days, before the plantlets reached the unviability and death phase, which is important from a practical perspective, and for further efficient and successful multiplication, rooting and acclimatization. In these processes, a single phytohormone may act as an inducer/stimulator or inhibitor at many timestamps. As tissues progress to successive stages of growth, they change their sensitivity to specific hormones [18], while the concentration of endogenous phytohormones depends on the rate of their biosynthesis, transport, conjugation and catabolism [76]. A majority of the endogenous phytohormones identified in the study belonged to stress-related phytohormones. The initiation of an in vitro culture by cutting the explants and placing them on a medium to start the next multiplication cycle is a cause of huge stress for a plant, as confirmed by the observed accumulation of stress hormones that gradually declined during the culture. This decrease was probably a sign of the culture stabilization. This happened first in the plants treated with red LED (R) light, in the first phase of multiplication, after 10 days. The levels of stress-related phytohormones may grow when the culture approaches its final stages, which may be associated with senescence [77]. In our culture, the most abundant phytohormones were benzoic and salicylic acids, which may have the reflected accumulation of phenolic compounds often observed in in vitro environments [78]. Also, benzoic acid cannot be considered only as a stress related hormone. It is an important building block (as well as other compounds like salicylic acid, aromatic cytokinins and secondary metabolites) [79]. This may explain its high amounts during the in vitro multiplication, especially under the RB LED light, where we observed the highest multiplication rate and quite high shoots. Contrary to that, ABA levels were very low, 500 times lower than those of benzoic acid, and never exceeded those in the starting material. As in the in vitro culture, the stomata are always open, and this can be explained by the lack of a need to control their movements [29]. Slightly higher accumulation of stress-related phytohormones could be indirectly responsible for the lowest multiplication factor and the shortest shoots observed in the plants grown under B LED light than under other light sources. However, JA and SA beyond defense (as well as gibberellins, ethylene, abscisic acid, and auxins) are also involved in plant growth and development. They take a part in processes such as vegetative growth, primary root growth, photosynthesis, respiration or senescence of leaf and whole plant [80,81]. Thus, an increase in JA and/or SA levels is not always linked with stress conditions.

Blue light can inhibit axillary shoot proliferation, however, this can be overcome by red light or cytokinin application. Light of a different wavelength is received by plants via specialized light receptors; phytochrome—red/far red light receptor [82], various blue light receptors such as cryptochrome [83] and phototropin [84], and the photoreceptor UV-B UV RESISTANECE LOCUS 8 [85] and ZEITLUPE [86]. They control different aspects of growth and morphogenesis [87]. Even though in the presence of exogenous plant regulators the indirect role of light in growth control may be imperceptible [70], our study confirmed high levels of stress-related phytohormones in the plants multiplied under B LED light. Growth inhibition may be related to the inhibition of the natural synthesis of cytokinins or ABA that acts antagonistically to cytokinins [18,70], however, our experiment revealed no effects of light on ABA tissue content. However, as ABA plays a regulatory role in organ induction, it is important for in vitro organogenesis [88]. ABA is involved in the induction of stomatal closure, inhibition of transpiration, and in stress-related gene expression. Lower concentrations of ABA enhance root hydraulic conductivity and the expression of genes of the inner plasma membrane—PIP protein aquaporins [89].

### 3.4. Cytokinins

In our experiments, cytokinins were the least numerous group of endogenous substances, which was due to the necessary addition of 5 µM of exogenous 6-benzyladenine (BA) to the multiplication medium. Their content during the culture, regardless of its phase and light quality, was always lower than in the starting material. Despite the endogenous presence of cytokinins in plants, many tissues isolated in vitro are not capable of synthesizing sufficient amounts of these substances to support growth. Total content of active endogenous cytokinins depends on multiple dynamic processes, such as their biosynthesis, formation or mobilization of storage forms (sequestered or O-glucosidic), and inactivation (N-glucosylation, alanine conjugation and removal of side chains by cytokinin oxidase) [18].

Exogenous cytokinins are often transformed in plant tissues into, for example, ring substitution products (ribosides, ribotides, N-glucosides) and side chain substitutions (O-glucosides) or cleavage products (adenine, adenosine, adenosine-5a-monophosphate) [90]. These metabolites and conjugates act as storage, transport or biologically inert forms of cytokinins responsible for the physiological and developmental plasticity of plants [71].

Research literature mentions a positive effect of exogenous BA, e.g., on the accumulation of IP and IPD, for example shoot organogenesis of *Petunia hybrida* leaf explants [91]. However, the role of endogenous hormone metabolism has not been fully explained for any in vitro developmental event [92]. Our study showed no effect of light quality on IP accumulation, even though R LED light clearly enhanced IPD levels.

Two main active cytokinins, zeatin and zeatin riboside, may be the hormones that determine the morphogenetic processes [93]. Zeatin is mainly present in living organisms as its trans isomer. Large amounts of cis-zeatin were detected e.g., in *Cicer* seeds [94], or seaweed [95], although the cis form is much less active than the trans one [70]. Our study identified both forms of zeatin (t-Z and c-Z). Their stimulating effect on gerbera growth was particularly visible in the plants grown under RB LED light, which showed the highest multiplication rate and high concentration of both zeatin forms at the end of the culture. Kinetin, the content of which was unaffected by the experimental conditions, is not considered a fully natural cytokinin and is believed to be a result of a structural rearrangement of DNA [70,96].

The most important factor controlling plant growth and development is proper auxin to cytokinin ratio. It is a key signal for the formation of cellular phenotype, as well as for the initiation and maintenance of cell division [70]. Many aspects of cell growth, differentiation and organogenesis in tissue and organ culture are controlled by cytokinin and auxin interaction. Auxins are, next to cytokinins, the most important group of hormones regulating growth and morphogenesis in in vitro cultures [18,70]. Our study demonstrated that light quality may be the major factor affecting auxin content. Auxin content in the plants grown under an Fl lamp was rising throughout the duration of the culture. Some reports indicate an involvement of auxin in the senescence. However, the mechanisms remain vaguely defined and the observations are contradictory [97]. Some suggest that auxin can inhibit senescence, whereas other experiments indicate that auxin can promote senescence [98]. In our experiment under B LED light auxin content initially dropped, although it then rose, which might be associated with the culture senescence.

Under R LED light, auxin level at the end of the culture was relatively low and gerbera shoots were the longest. We therefore concluded that red LED light accelerated stress relief (lower content of stress-related phytohormones), which allowed the plants to grow faster (higher shoots), yet also promoted earlier senescence (lowered auxin levels). Low concentration of auxins together with high levels of cytokinins is often beneficial at the second stage of the culture during shoot propagation, even though in some cases cytokinins alone are sufficient [70]. 

### 3.5. Auxins

Among exogenous auxins added to the medium, the active, fairly unstable form of IAA is particularly sensitive to light (wavelength, light intensity and photoperiod) [13]. Its accelerated metabolism and photodegradation may be to the greatest extent affected by the wavelength close to the UV and blue range [70]. Its concentrations are usually high in young, rapidly growing organs, and its biosynthesis is the most intense in the meristematic regions. These high levels decrease with the plant age [70]. Our analyses of endogenous IAA did not confirm this pattern at the stage of shoot propagation in vitro, which might be due to the type of plant material analyzed, as we investigated the entire shoots and not only the most juvenile tissues. We also did not confirm the degrading effect of blue light on the content of endogenous IAA. The level of endogenous free auxins depends of the rate of their anabolism, catabolism, transport and conjugation [18]. IAA is stored in the cells in the form of conjugates that allow for stabilizing the levels of free auxin and metabolizing its excess. Conjugated auxin is protected against oxidative degradation and can be released by appropriate enzymes [51,70]. The contents of the substances belonging to the auxin group determined in our study remained within a relatively narrow range, and the discrepancies between them were small, which was not the case in the other groups of growth regulators. The identified forms of auxins are constantly bound and released, used and synthesized by the plant during its growth and development. Auxin precursors, which may also have auxin-like properties, can sometimes replace IAA and be more effective than an auxin alone in stimulating growth or inducing organized development [18]. The content of IBA, lower for all treatments and stages of the culture than in the starting material, differed from the general tendency. IBA is more stable than IAA and its polar transport is more efficient [76].

### 3.6. Gibberellins

Gibberellins affect the rate of shoot proliferation and can also increase shoot length, especially when the shoots are short due to high cytokinin levels. So far, about 90 natural gibberellins have been described [18], most of which are precursors or inactivated forms. Just a few of them are biologically active [99]. In our study, blue LED light (B and RB) lowered total gibberellin content at the end stage of shoot propagation. The most abundant gibberellin was GA_6_, while GA_7_, considered one of the most active gibberellins, was relatively scarce. The greatest shoot elongation due to accumulation of gibberellins was observed in plants multiplied under red LED light. Light and its parameters (such as wavelength or photoperiod) regulate gibberellin metabolism in plants [18]. For example, phytochrome B mediates the photoperiodic regulation of gibberellin production in potatoes [100]. Plant growth may be inhibited when the light spectrum contains considerable amounts of blue or UV light that promote gibberellin biosynthesis. Such growth inhibition (low multiplication rate and shorter shoots) was visible in gerbera shoots, especially under B light, where total gibberellin content at the last stage of the culture was the highest. George et al. [70] reported that a gibberellin inhibitor may stimulate growth of *Haplopappus gracilis* callus and cell suspensions.

## 4. Materials and Methods

### 4.1. Plant Material and Growth Conditions

Plant material used in the experiment involved in vitro multiplied axillary shoots of *Gerbera jamesonii* Bolus ex Hook. f. cv. ‘Big Apple’. It is a variety obtained by Schreurs Holland B.V. company (De Kwakel, Holland), sourced from Teresa Foszczka in vitro Laboratory (Rzgów, Poland). Plant material was collected and cultured on Murashige and Skoog [101] medium (MS) supplemented with sucrose in concentration of 30 g·dm^−3^, 5 µM 6-benzyladenine (BA) (Duchefa Biochemie, Haarlem, The Netherlands), and 0.5% BioAgar (Biocorp, Warsaw, Poland), at pH 5.7. 

The experiment included the second stage of clonal propagation, i.e., the propagation of axillary shoots. Single axillary shoots—rosettes devoid of leaf blades, were placed on the medium and multiplied in 200 mL glass jars covered with polyvinyl chloride caps with an air orifice (MZ Forma, Łódź, Poland). Five gerbera axillary shoots were planted in each jar, and the entire micropropagation cycle lasted for six weeks (40 days) (subcultures). The conditions in a growth chamber with 16/8 h photoperiod (day/night) were: temperature 23/21 ± 1 °C (day/night) and 80% relative humidity. The experimental factor was light of different quality. There were three spectrum treatments: red (670 nm) and blue (430 nm) light (100% R and 100% B, respectively), and the combination of these lights in the 7:3 ratio (RB). These parameters were provided by a Solid State Lighting (light-emitting diodes) system (SSL LED) and were set using a BTS256 spectrometer (Gigahertz-Optik, Germany) and a LI-250A light meter equipped with a Q 50604 sensor (LI-COR, Lincoln, NE, USA). The settings were controlled using the DXM-512 digital protocol. A fluorescent lamp (Fl) (Philips TL-D 36W/54), emitting white light, served as a control [28]. In all treatments the photosynthetic photon flux density (PPFD) was 35 μmol m^−2^ s^−1^.

### 4.2. Experimental Design and Data Collection

The chemical analyses investigated the level of various PhRCs in plant tissues during and after the shoot multiplication culture. The samples were collected every 10 days. We also collected a control sample, i.e., axillary shoots cut and prepared to be placed on the medium (starting material). 

After completion of the shoot multiplication cycle (40th day of the culture), we performed a biometric evaluation of the shoots and determined the fresh and dry weight of the plants.

The content of endogenous phytohormones in gerbera tissues was determined with an ultra-high performance liquid chromatography (UHPLC) in 0.5 g samples of developed upper parts of the multiplied gerbera (shoots and leaves). The substance determination was carried out according to Dziurka et al. [102]. Phytohormones were extracted from the lyophilized plant material to an aqueous solution of formic acid in methanol, according to Dobrev and Kaminek [103]. The stable isotope-labeled internal standard mixture was added at this stage. Following centrifugation, the supernatants were evaporated under nitrogen, and then the residues were dissolved in a 5% methanol solution in 1 M formic acid and purified on Bond Elut Plexa PCX columns. The phytohormone-containing fractions were eluted with a methanol/acetonitrile solution and 5% ammonia in a methanol/acetonitrile solution. After evaporation, the extracts were dissolved in acetonitrile. The samples were determined using a UHPLC (Agilent 1260, Agilent Technologies, Waldbronn, Germany) with a tandem quadrupole mass spectrometry detector (Agilent 6410, Agilent Technologies, Santa Clara, CA, USA). An analytical column (Ascentis Expres RPAmide 2.7 μm, 2.1 mm × 150 mm, Supelco, Bellefeonte, PA, USA) with a linear gradient of 0.01% formic acid in water and 0.01% formic acid in acetonitrile were used. The phytohormones were detected in a positive ion mode after electrospray ionization (ESI) under atmospheric pressure. Data were acquired in multiple reaction monitoring (MRM) mode. Internal standard mixture consisted of [^2^H_5_]indole-3-acetic acid methyl ester (MeIAA-D5), [^2^H_5_]indole-3-acetic acid (IAA-D5), [^15^N_4_]kinetin (K-N15,), [^15^H-N_4_]dihydrozeatin (DHZ-N15), [^2^H_5_]trans-zeatin riboside (t-ZR-D5), [^2^H_2_] gibberellin A4 (GA4-D2), [^2^H_2_] gibberellin A_5_ (GA5-D2), [^2^H_2_] gibberellin A6 (GA6-D2), [^2^H_2_]gibberellin A_1_ (GA1), [^2^H_2_]gibberellic acid (GA3-D2), [^2^H_2_]gibberellin A_8_ (GA8-D2), [^2^H_4_]salicylic acid (SA-D4), [^2^H_6_]cis, trans-abscisic acid (ABA-D6). All standards, except for [^2^H_5_] JA (JA-D5) supplied by CND Isotopes (Quebeck, Canada) and [^2^H_5_] dinor-12-oxo-OPDA (dinor-12-oxo-OPDA-D5) supplied by Cayman Chem. Comp. (Ann Arbor, MI, USA), were from OlChemim (Olomouc, Czech Republic) at the highest available purity. The results were referenced to the calibration curve for pure standards, including the internal isotopic standards recovery. Further technical details are given in the Supplementary Materials (Tables S2 and S3). To obtain information on the plant morphometric response, multiplication rate, plantlet height and the number of new leaves per shoot were assessed based on morphometric observations. We also determined the fresh weight of multiplied plants, and measured their dry weight by drying 200 mg plant samples in a laboratory dryer (65 °C, Sanyo MOV-112S) to constant weight.

### 4.3. Statistical Analysis

The culture of multiplied shoots involved five repetitions per treatment, with five explants each (in total, 100 explants). We obtained a different average number of plants on each light quality treatment, consecutively: 178 from B LED, 320 from RB LED, 227 from R LED and 205 from Fl light (930 plants in total) (Figure 1). From obtained plants, samples were taken for analysis in the conducted experiments. To analyze the endogenous phytohormone content in gerbera tissues during the entire experiment, five samples were taken randomly from each combination, making it a total of 85 samples of plant material with a total weight of 42.5 g. All collected data were subjected to statistical analysis using Statistica software version 13 (TIBCO Software Inc., Palo Alto, CA, USA). The effects of the treatment were tested for significance using analysis of variance (ANOVA). The Duncan post hoc multiple range test was used to separate significantly different means and to provide homogeneous groups for the means (at *p* ≤ 0.05). The Dunnett’s test was used to compare the content of endogenous growth regulators in gerbera tissues in the experimental and starting material. To compare the content of growth regulators belonging to different groups of substances, logarithmic averaging of the data was used.

## 5. Conclusions

Our studies for the first time analyzed the plant tissue response to a different quality of light at subsequent stages of *Gerbera* axillary shoot culture in aspects of endogenous PhRC content. Our results broaden the knowledge on the mechanisms of PhRC transformation in plants cultivated in vitro and their relationship with the plant morphometric response. In this context they demonstrated that modern LED lighting can be successfully used in this clonal propagation stage. LED light does not induce unfavorable disturbances in PhRC balance, and it even seems to alleviate in vitro stress, when compared with the traditional method of culture lighting. The stress was the most effectively reduced under red LED. Red LED wavelength (R and RB) lowered tissue auxin levels, and the process was quicker under red only (R) than red and blue light (RB). Blue LED light (B) lowered the shoot multiplication rate and their height, while the highest content of gibberellins at the last stage of the culture was observed under this light.

## Figures and Tables

**Table 1:** Total PhRC content from the stress-related phytohormone group in gerbera tissue samples during the culture.

Days of Culture	Light Quality	Cytokinins	Auxins	Gibberellins	Stress-Related Acids
Starting Material		0.84 ± 0.05	8.12 ± 0.83	3.42 ± 0.51	360.85 ± 33.24
10	B	0.46 ± 0.06	6.42 ± 0.65	8.02 ± 1.04	119.36 ± 12.20
	RB	0.42 ± 0.03	5.34 ± 0.74	9.22 ± 1.73	83.79 ± 5.78
	R	0.48 ± 0.06	5.52 ± 0.51	9.65 ± 1.06	77.43 ± 4.01
	Fl	0.40 ± 0.02	5.62 ± 0.52	7.42 ± 0.85	164.85 ± 16.05
20	B	0.41 ± 0.04	9.08 ± 0.58	10.11 ± 2.47	247.63 ± 35.33
	RB	0.42 ± 0.02	8.56 ± 0.86	9.75 ± 1.32	217.31 ± 25.59
	R	0.45 ± 0.03	7.74 ± 0.65	15.83 ± 5.50	98.29 ± 6.42
	Fl	0.38 ± 0.06	6.11 ± 1.65	8.73 ± 3.30	119.49 ± 15.43
30	B	0.41 ± 0.01	8.83 ± 1.31	8.98 ± 3.05	207.13 ± 8.58
	RB	0.49 ± 0.02	11.15 ± 1.35	10.28 ± 3.04	200.46 ± 38.16
	R	0.44 ± 0.05	6.77 ± 0.64	9.22 ± 3.52	155.23 ± 16.80
	Fl	0.42 ± 0.02	7.69 ± 1.55	6.57 ± 1.08	227.00 ± 41.40
40	B	0.45 ± 0.02 a	9.09 ± 0.66 b	8.37 ± 1.87 b	198.02 ± 42.21 ab
	RB	0.42 ± 0.03 a	5.70 ± 0.48 a	9.34 ± 1.76 b	155.41 ± 30.13 a
	R	0.42 ± 0.04 a	5.43 ± 0.77 a	3.91 ± 0.58 a	160.49 ± 17.99 a
	Fl	0.44 ± 0.03 a	8.18 ± 0.80 b	4.40 ± 0.62 a	226.65 ± 52.95 b

B—100% blue LED (430 nm); RB—combination of red (70%) and blue (30%) LED; R—100% red LED (760 nm); Fl—control fluorescence Philips TK-D 36W/54 lamps. The ANOVA analysis was conducted for plant material at the end of the culture (after 40 days) to compare the results with the morphometric parameters. Means ± standard deviations within a column followed by the same letter (a, b) are not significantly different according to Duncan’s multiple range test at *p* ≤ 0.05. Data for particular phytohormones and related substances concentrations are given in Supplementary Materials (Table S1).

## Data Availability

Data are contained within the article and Supplementary Materials.

## Supplementary Materials

The following supporting information can be downloaded at: https://www.mdpi.com/article/10.3390/molecules27061804/s1, Table S1: Mean content (pmol/g) of determined endogenous hormones and related compounds (PhRC) in starting material and in the stages of culture (after 10, 20, 30 and 40 days) in relation to treatment with light of different qualities. Table S2: The optimized mass spectrometry parameters for phytohormone quantitation. The following conditions were optimal for the analysis: capillary voltage 4 kV, gas temperature 350 °C, gas flow 12 L/min, and nebulizer pressure of 35 psi. The measurements were conducted by multiple reaction monitoring (MRM) in positive polarity. MassHunter software was used to control the LC-MS/MS system and data analysis. For MRM parameters, MassHunter Optimizer was used. In case of internal standards (ISTD) in parenthesis are given their quantities. Table S3: Validation parameters of phytohormone estimation method.

## Sample Availability

Samples of the compounds are not available from authors.
