# Supplementary material for: Changes in Endogenous Phytohormones of Gerbera jamesonii Axillary Shoots Multiplied under Different Light Emitting Diodes Light Quality
Source: Molecules. 2022 Mar 10;27(6):1804. doi: 10.3390/molecules27061804 (PMC8950344; doi:10.3390/molecules27061804)
Supplement: Supplementary file 1 [file molecules-27-01804-s001.zip › molecules-1601244-supplementary.pdf]

Supplementary Material for:

Changes in endogenous phytohormones of *Gerbera jamesonii* axillary shoots multiplied under different Light Emmiting Diodes light quality

Monika Cioć [1,*], Michał Dziurka [2] and Bożena Pawłowska [1]

[1] Department of Ornamental Plants and Garden Art, Faculty of Biotechnology and Horticulture, University of Agriculture in Kraków, 29 Listopada 54, 31-425 Kraków, Poland; monika.cioc@urk.edu.pl
[2] Department of Developmental Biology, The Franciszek Górski Institute of Plant Physiology, Polish Academy of Sciences, Niezapominajek 21, 30-239 Kraków, Poland; m.dziurka@ifr-pan.edu.pl
* Correspondence: monika.cioc@urk.edu.pl

Table S1. Mean content (pmol/g) of determined endogenous hormones and related compounds (PhRC) in starting material and in the stages of culture (after 10, 20, 30 and 40 days) in relation to treatment with light of different qualities.

Days	Light quality	CYTOKININS												
		**IPD**	**IP**	**t-ZR**	**DH-ZR**	**DH-Z**	**t-Z**	**t-Z7G**	**t-ZOG**	**c-ZR**	**c-Z**	**KR**	**K**	**Σ**
Starting material		13.08	49.86	12.85	3.75	209.87	22.75	3.24	4.36	25.58	1.40	107.88	386.17	840.79
10	B [1]	3.06	44.81	13.36	4.39	234.79	24.08	2.93	4.16	17.93	0.66	59.69	45.98	455.83
	RB	8.66	37.26	12.30	3.24	225.75	24.24	2.63	4.45	16.11	0.78	55.15	30.84	421.39
	R	6.96	50.00	13.73	5.44	260.72	22.08	3.2	4.60	12.39	0.52	61.10	43.11	483.86
	Fl	7.80	37.24	12.28	3.35	219.38	20.03	1.81	3.71	18.17	0.55	45.84	26.47	396.61
20	B	3.26	43.61	13.66	3.49	231.26	24.73	2.75	3.13	12.78	0.96	44.38	27.07	411.07
	RB	5.95	42.58	13.06	3.98	234.38	23.03	2.66	4.01	15.48	0.69	53.23	34.69	433.75
	R	4.29	53.69	12.91	4.51	256.86	28.37	3.10	4.81	15.63	0.66	50.27	14.08	449.17
	Fl	4.57	27.75	9.93	4.15	232.72	22.42	2.37	3.49	18.59	0.54	44.92	11.49	382.94
30	B	7.70	44.33	13.30	4.04	219.74	22.57	2.67	3.80	9.99	1.30	43.28	38.57	411.29
	RB	10.48	59.95	14.29	4.26	259.84	27.85	2.99	4.59	13.57	1.01	46.61	49.26	494.70
	R	12.70	58.58	11.84	4.35	238.55	25.26	2.89	4.53	14.21	0.75	46.02	24.70	444.38
	Fl	17.22	45.77	12.74	3.32	214.60	24.90	3.23	4.48	14.84	1.30	43.30	35.10	420.80
40	B	8.71	63.14	13.07	5.77	245.26	26.68	2.92	3.75	8.01	1.23	45.06	30.57	454.18
	RB	8.85	58.73	13.01	2.48	226.76	24.48	3.12	4.25	8.69	0.82	43.72	28.69	423.61
	R	21.29	62.30	11.13	6.04	219.84	21.52	4.97	3.84	7.36	0.47	39.19	23.20	421.16
	Fl	8.22	60.00	15.03	4.71	241.28	20.82	2.85	5.61	10.10	1.18	45.32	28.38	443.49
Mean:		12.18	45.44	12.48	4.26	237.66	24.20	3.39	4.37	12.98	0.89	50.15	30.38	438.82

		AUKSINS							
		**IAA-Carb**	**IBA**	**IAA**	**IAA-Glut**	**IAA-ASA**	**oxo-IAA**	**IAA-Met**	**Σ**
Starting material		2761.83	784.78	1603.11	489.35	1602.69	474.76	405.94	8122.45
10	B	1679.04	315.64	1100.37	1304.08	611.61	1211.73	202.04	6424.51
	RB	1384.39	296.56	952.46	1215.72	452.18	872.39	162.86	5336.55
	R	1199.72	217.54	1479.46	920.31	488.24	989.22	221.83	5516.33
	Fl	1638.82	245.64	928.05	1110.24	403.60	1107.57	186.37	5620.28

20	B	3232.47	310.13	1476.28	1977.78	793.21	1049.45	243.23	9082.54
	RB	1826.89	277.10	1187.32	1305.63	549.77	1046.07	203.27	6396.04
	R	2191.37	286.27	1462.44	2145.53	537.12	844.99	274.20	7741.92
	Fl	1429.73	249.09	1141.74	1624.36	540.32	890.22	230.42	6105.88
30	B	2669.82	290.93	1515.15	1303.28	1835.32	918.82	298.40	8831.72
	RB	3457.48	337.94	1638.63	2372.35	234.55	623.16	401.31	11145.41
	R	2241.33	444.69	1372.19	1034.28	730.44	612.06	335.12	6770.11
	Fl	3801.09	308.39	1335.70	87.63	1148.42	679.45	330.92	7691.61
40	B	2987.57	223.27	1551.01	2296.52	704.31	954.11	374.11	9090.88
	RB	1950.54	209.27	1179.67	967.90	626.37	424.72	338.67	5697.15
	R	2502.13	319.81	1073.86	138.60	641.61	387.84	368.30	5432.14
	Fl	3179.52	238.87	1738.93	996.75	760.65	808.95	454.42	8178.08
Mean:		2500.41	326.98	1333.49	1229.99	1359.07	799.78	308.64	8240.94

GIBBERELLINS

		GA_1	GA_3	GA_4	GA_5	GA_6	GA_7	GA_8	GA_9	GA_{20}	Σ
Starting material		303.60	981.61	104.69	35.37	1571.99	17.97	297.23	66.52	42.52	3421.49
10	B	378.79	935.80	65.86	104.96	5664.88	7.04	721.08	59.15	79,32	8016.88
	RB	259.79	1388.30	99.55	89.19	6628.64	9.78	596.86	56.53	91.18	9219.83
	R	429.36	737.36	117.50	140.98	7281.39	10.02	661.42	81.32	193.65	9653.01
	Fl	319.63	995.04	94.83	118.20	5165.71	6.75	557.43	56.74	109.82	7424.15
20	B	270.48	1771.42	95.22	66.98	72.51.86	6.32	517.33	81.30	44.16	10105.09
	RB	331.61	1165.58	94.60	104.06	63.98.50	7.98	610.82	67.01	103.63	8883.79
	R	383.56	403.55	132.48	44.09	13957.83	8.93	682.95	77.69	134.66	15825.75
	Fl	354.66	1573.84	92.84	69.17	5910.35	7.27	467.28	75.39	183,47	8734.27
30	B	201.29	1273.16	108.90	16.45	6863.17	5.96	406.65	63.89	37.74	8977.21
	RB	351.75	961.64	205.73	23.27	8081.53	7.06	524.31	77.46	47.11	10279.86
	R	195.57	687.26	10.63	39.24	7577.39	10.43	477.60	64.09	60.77	9222.98
	Fl	193.91	975.65	107.13	14.86	4671.94	6.39	491.71	72.47	35.76	6569.82
40	B	342.38	2331.41	87.98	19.2	5321.96	4.85	151.44	73.94	41.12	8374.39
	RB	532.51	1775.08	121.72	23.75	6484.09	6.86	289.84	64.23	41.88	9339.96
	R	169.99	1008.90	457.80	13.35	2039.72	6.44	112.53	63.59	35.41	3907.71
	Fl	447.09	1076.39	89.53	26.72	2117.72	6.11	512.76	77.57	49.50	4403.38
Mean:		351.25	1367.48	261.83	77.16	7998.78	7.64	416.80	68.93	114.53	9866.73

STRESS-RELATED PHYTOHORMONES

		BeA (benzoic acid)	SA	12-oxo-PDA	JA	JA-Met	ABA	ABA-GLU	Σ
Starting material		180809.46	164168.68	11430.20	3118.68	34.13	575.67	709.33	360846.12
10	B	89419.60	22201.59	4753.29	2442.93	37.26	242.52	264.80	119361.99
	RB	54875.13	21555.01	4296.79	2550.14	31.59	306.89	177.12	83792.67
	R	47984.71	23990.43	9655.75	1381.81	39.31	104.23	270.18	77426.43
	Fl	133375.77	23389.63	5114.17	2315.04	35.87	287.90	328.33	164846.72
20	B	168154.43	65449.35	6941.15	6547.09	35.20	219.05	280.78	247627.06
	RB	98761.93	31317.20	4952.23	3047.40	35.85	232.12	264.24	138610.97
	R	41612.05	48349.69	4996.96	2882.71	37.39	166.88	244.38	98290.07
	Fl	58524.75	48408.66	8580.79	3419.09	34.82	189.08	330.25	119487.44
30	B	149975.06	45047.27	8537.23	2819.50	34.92	194.17	520.15	207128.29
	RB	127428.48	59483.98	10315.74	2429.51	42.30	215.17	544.76	200459.94
	R	106946.51	41061.65	5390.54	1281.32	36.19	185.71	326.19	15228.10
	Fl	149184.30	55962.46	10836.74	10119.09	33.79	210.19	654.35	227000.91
40	B	154511.27	23235.28	16189.64	3229.90	37.95	286.31	526.26	198016.60
	RB	119965.04	17720.18	15056.34	1702.13	37.64	200.58	726.69	155408.60
	R	111105.89	32909.87	13802.33	1773.26	33.44	198.82	664.97	160488.57
	Fl	140235.15	72489.29	11056.35	2133.82	38.10	251.11	441.96	226645.79
	Mean:	104883.24	45104.74	9922.70	5700.20	36.95	205.56	451.90	162526.74

[1] B—100% blue LED (430 nm); RB—combination of red (70%) and blue (30%) LED; R—100% red LED (670 nm); Fl—control. fluorescence Philips TK-D 36W/54 lamps.

Table S2. The optimized mass spectrometry parameters for phytohormone quantitation. The following conditions were optimal for the analysis: capillary voltage 4 kV, gas temperature 350 °C, gas flow 12 l/min, and nebulizer pressure of 35 psi. The measurements were conducted by multiple reaction monitoring (MRM) in positive polarity. MassHunter software was used to control the LC-MS/MS system and data analysis. For MRM parameters, MassHunter Optimizer was used. In case of internal standards (ISTD) in parenthesis are given their quantities..

Compound		Type of ion	Quantifier transition (precursor/product ions)	Fragmentor voltage (V)	Collision energy (V)	MRM Start Time (min.)
t-Z7G		$[M+H]^+$	382.1/220.1	122	17	1
t-Z		[M+H]+	220.2/136.3	85	9	
t-ZOG		$[M+H]^+$	382.1/202.1	142	17	
DHZ-N15	ISTD (10 pmol)	[M+H]+	226.2/152	124	18	
DH-Z		[M+H]+	222.2/136	124	18	
c-Z		[M+H]+	220.2/136.3	85	9	
K-N15	ISTD (10 pmol)	[M+H]+	220.1/192.3	90	9	4.7
K		[M+H]+	216.1/188.3	90	9	
GA8-D2	ISTD (10 pmol)	$[M-H_2O+H]^+$	321.2/303.1	150	5	
GA8		$[M-H_2O+H]^+$	319.3/257.2	102	9	
oxo-IAA		$[M+H]^+$	192.2/146.1	54	9	
t-Z-R-D5	ISTD (10 pmol)	$[M+H]^+$	357.3/225.2	116	17	5.9
t-Z-R		$[M+H]^+$	352.2/220.3	120	9	
DH-Z-R		$[M+H]^+$	354,2/222,1	124	14	
c-Z-R		$[M+H]^+$	352.2/220.3	120	9	
IP		$[M+H]^+$	204.1/148.3	90	9	7.3
IAA-ASA		$[M+H]^+$	291.2/130.1	54	25	
BeA-D4	ISTD (500 pmol)	$[M+H]^+$	128.1/84.1	61	13	
BeA		$[M+H]^+$	123.1/79.1	56	13	
KR		$[M+H]^+$	204,1/148,3	90	9	9.1
IAA-Glu		$[M+H]^+$	305.2/130.1	58	29	
GA3-D2	ISTD (10 pmol)	[M-H2O+H]+	331.2/314.1	100	14	11
GA3		[M-H2O+H]+	329.3/311.3	100	14	
GA1-D2	ISTD (10 pmol)	$[M-H_2O+H]^+$	333.3/287.2	58	9	
GA1		[M-H2O+H]+	331.3/285.3	100	14	
IAA-Carb		$[M+H]^+$	162.2/118.1	58	9	
IAA-D5	ISTD (100 pmol)	[M+H]+	181.1/135.1	38	14	
IAA		[M+H]+	176.1/130.3	51	9	
SA-D4	ISTD (500 pmol)	[M+H]+	143.2/125.2	80	14	11.3
SA		[M+H]+	139.2/121.2	80	14	
ABA-GLU		$[M-H_2O+H]^+$	409.2/247.1	104	14	12.0
GA6-D2	ISTD (10 pmol)	$[M-H_2O+H]^+$	331.3/115.1	96	5	13.2
GA6		[M-H2O+H]+	329.3/283.3	104	14	
IPD		$[M+H]^+$	336.2/204.1	124	14	
ABA-D6	ISTD (30 pmol)	[M-H2O+H]+	253.4/191.3	80	14	17.5
ABA		[M-H2O+H]+	247.4/187.2	80	14	
GA5-D2	ISTD (10 pmol)	$[M-H_2O+H]^+$	287.3/115.0	96	5	18.0
GA5		$[M-H_2O+H]^+$	285.1/115.0	96	5	
GA20		$[M-H_2O+H]^+$	287.3/115.0	96	5	
IAA-Met-D5	ISTD (100 pmol)	[M+H]+	195/134	54	9	18.8
IAA-Met		[M+H]+	190/130	54	9	
JA-D5	ISTD	$[M+H]^+$	216.3/153.2	80	5	19.5

Compound		Type of ion	Quantifier transition (precursor/product ions)	Fragmentor voltage (V)	Collision energy (V)	MRM Start Time (min.)
	(100 pmol)					
JA		$[M+H]^+$	211.3/151.2	80	14	
GA7		$[M-H_2O+H]^+$	313.2/223.1	104	14	20.9
GA4-D2	ISTD (10 pmol)	$[M-H2O+H]^+$	317.3/271.2	88	9	
GA4		$[M-H2O+H]^+$	315.3/269.3	100	14	
JA-Met		$[M+H]^+$	225.3/151.2	58	5	22.51
GA9		$[M-H2O+H]^+$	271.3/225.2	136	13	23.1
dinor-12-oxo-OPDA-D5	ISTD (10 pmol)	$[M+H]^+$	270.3/252.2	84	5	23.72
12-oxo-PDA		$[M+H]^+$	293.3/275.2	68	9	25.54

Table S3. Validation parameters of phytohormone estimation method.

Compound	Calibration curve y =ax + b		R^2	calibration levels	linearity range (ng/ml)	LOD (ng/ml)	LOQ (ng/ml)	M (mol/g)	LOD (pmol/ml)	LOQ (pmol/ml)	Recovery† (%)	Matrix† effect (%)
	a	b										
t-Z7G	188.58	-142.26	0.9994	6	1.7 to 76.7	0.05	0.15	381.4	0.13	0.39	99 (2)*	83 (9)
t-Z	58.01	-61.13	0.9997	6	2.8 to 141.5	0.79	2.40	219.3	3.60	10.95	109 (5)	90 (17)
t-ZOG	154.29	-299.09	0.9971	6	1.8 to 84.4	0.05	0.15	381.4	0.13	0.39	100 (2)	83 (10)
DH-Z	67.28	-133.55	0.9980	6	4.7 to 219.4	0.79	2.40	221.3	3.57	10.85	101 (2)	83 (9)
c-Z	114.85	-25.84	0.9999	6	2.9 to 132.5	0.22	0.67	219.3	1.00	3.06	101 (2)	83 (9)
KIN	25.53	-5.03	0.9997	6	9.4 to 434.5	2.25	6.83	215.2	10.45	31.73	97 (3)	88 (6)
oxo-IAA	57.44	-119.60	0.9995	6	9.1 to 421.9	0.71	2.14	191.2	3.71	11.19	84 (2)	75 (7)
GA8	1.13	-5.41	0.9999	6	27.4 to 1265.8	3.81	11.53	364.4	10.46	31.64	92 (20)	75 (11)
t-Z-R	56.33	-6.22	0.9992	6	3.2 to 149.4	0.28	0.86	351.4	0.80	2.45	103 (3)	76 (8)
DH-Z-R	132.17	-27.25	0.9999	6	1.4 to 65.8	0.14	0.44	353.4	0.40	1.25	102 (3)	78 (8)
c-Z-R	205.97	-56.38	0.9991	6	1.3 to 60.2	0.51	1.56	351.4	1.45	4.44	108 (2)	79 (5)
IAA-ASA	3.29	-18.68	0.9994	6	9.3 431.5	3.00	9.10	290.3	10.33	31.35	88 (2)	79 (7)
IP	309.68	-730.26	0.9991	6	9.8 to 451.2	0.17	0.53	203.3	0.84	2.61	102 (3)	82 (8)
BeA	2.39	5.88	0.9999	6	27.6 to 1278.2	15.16	45.95	122.1	124.14	376.27	77 (5)	43 (10)
IAA-GLUT	10.45	-131.79	0.9954	6	9.2 to 425.1	2.60	7.88	304.3	8.54	25.89	89 (2)	78 (7)
KR	27.52	2.59	0.9998	6	4.6 to 210.9	0.82	2.48	347.3	2.36	7.14	103 (3)	79 (8)
SA	5.61	-24.43	0.9996	6	11.6 to 538.5	4.31	13.07	138.1	31.20	94.63	79 (5)	45 (9)
IAA-Carb	3.15	4.91	1.0000	6	29.6 to 1369.6	12.28	37.20	161.2	76.20	230.83	76 (2)	45 (8)
IAA	27.95	-24.85	0.9998	6	6.4 to 296.9	2.63	7.98	203.2	12.94	39.26	74 (2)	49 (7)
GA3	0.78	2.21	1.0000	6	29.2 to 1351.0	14.93	45.25	346.4	43.10	130.64	94 (20)	61 (20)
GA1	4.31	-4.64	1.0000	6	27.4 to 1265.8	5.20	15.75	348.4	14.93	45.21	91 (1)	106 (14)
ABA-GLU	32.35	212.28	0.9996	6	18.2 to 843.9	0.32	0.96	426.5	0.75	2.25	103 (4)	85 (10)
GA6	0.41	-9.01	0.9982	6	54.7 to 2531.6	12.01	36.40	346.4	34.67	105.09	98 (15)	88 (18)
IPAD	860.86	-969.53	0.9990	6	3.6 to 168.1	0.08	0.24	335.4	0.24	0.72	109 (5)	94 (17)
ABA	9.95	-17.16	0.9982	6	9.2 to 427.7	1.78	5.38	264.3	6.73	20.35	105 (4)	87 (10)
GA5	8.81	-5.62	0.9996	6	9.1 to 421.9	0.90	2.74	330.4	2.72	8.29	100 (5)	106 (14)
GA20	15.375	-48.458	0.9997	6	35 to 702	2.28	6.9	332.4	6.86	20.76	104 (4)	106 (14)
IAA-Met	8.99	-23.69	0.9983	6	9.1 to 421.9	0.78	2.38	189.2	4.12	12.58	79 (2)	59 (7)
JA	4.26	17.48	0.9994	6	18.2 to 843.9	1.74	5.27	210.3	8.27	25.06	97 (18)	87 (18)
IBA	164.48	-312.42	0.9998	6	12.8 to 592.8	0.96	2.91	203.2	4.72	14.32	85 (4)	63 (7)
GA7	2.47	10.10	0.9996	6	18.2 to 843.9	3.07	9.30	330.4	9.29	28.15	99 (10)	87 (12)
GA4	11.45	16.84	0.9998	6	9.1 to 421.9	1.59	4.81	332.4	4.78	14.47	99 (1)	82 (9)

Compound	Calibration curve y =ax + b a	b	R^2	calibration levels	linearity range (ng/ml)	LOD (ng/ml)	LOQ (ng/ml)	M (mol/g)	LOD (pmol/ml)	LOQ (pmol/ml)	Recovery† (%)	Matrix† effect (%)
JA-Met	9.71	13.06	0.9999	6	9.4 to 434.5	1.82	5.51	224.3	8.11	24.57	87 (9)	85 (12)
GA9	2.68	10.67	0.9999	6	18.2 to 843.9	0.54	1.64	316.4	1.71	5.18	108 (9)	104 (9)
12-oxo-PDA	7.03	6.18	0.9999	6	18.2 to 843.9	2.42	7.33	292.4	8.28	25.07	126 (17)	109 (17)

LOD – limit of detection 3.3×S/a; LOQ limit of quantitation 10×S/a; where S – standard deviation of peek area in calibration curve, a- sensitivity (slope of calibration curve).

† The recovery and recovery influenced by matrix effects (matrix effect) were examined by comparing the MS signals (peak areas) for three sets of samples: (a) phytohormones solutions in acetonitrile, (b) phytohormones in sample extracts, and (c) sample extracts spiked with phytohormones before extraction. For the recovery estimation, peak areas in the c and b sets were compared. For matrix effect, the peak areas for the b and a sample sets were used.

* Values in parenthesis are standard deviation (n =3).
